# Relation between diagnosis of atheromatous plaque from orthopantomographs and cardiovascular risk factors. A study of cases and control subjects

**DOI:** 10.4317/medoral.20183

**Published:** 2015-11-22

**Authors:** Cristina Barona-Dorado, Carmen Gutierrez-Bonet, Isabel Leco-Berrocal, Fernando Fernández-Cáliz, José-María Martínez-González

**Affiliations:** 1 Associate Lecturer in Buccal Surgery. Faculty of Dentistry, Complutense University of Madrid, Spain. Assistant Director of Master’s Program in Buccal Surgery and Implant Dentistry, Virgen de la Paloma Hospital, Madrid, Spain; 2 Resident Lecturer, Master’s Program in Buccal Surgery and Implant Dentistry, Virgen de la Paloma Hospital, Madrid, Spain; 3 Associate Lecturer in Buccal Surgery. Faculty of Dentistry, Complutense University of Madrid, Spain. Lecturer, Master’s Program in Buccal Surgery and Implant Dentistry, Virgen de la Paloma Hospital, Madrid, Spain; 4 Senior Lecturer in Maxillofacial Surgery, Faculty of Dentistry, Complutense University of Madrid, Spain. Director of the Master’s Program in Buccal Surgery and Implant Dentistry, Virgen de la Paloma Hospital, Madrid, Spain

## Abstract

**Background:**

In recent years the use of orthopantomography has been proposed as a low-cost, reliable and non-invasive diagnostic medium for detecting atheromatous plaque. The purpose of this study was to correlate the presence of carotid calcifications (atheroma) in orthopantomographs with specific risk factors for cerebrovascular accidents (previous cerebrovascular accidents, arterial hypertension, and diabetes).

**Material and Methods:**

The methods used in this observational study of cases and control subjects followed STROBE (Strengthening the Reporting of Observational studies in Epidemiology) recommendations. The study analyzed a total of 1,602 panoramic radiographs taken for dental diagnostic purposes between January 2010 and February 2014. The main variables analyzed were the incidence of atheromatous plaque and other cardiovascular risk factors. Epidat 3.1 statistical software was used to determine minimum sample sizes and the results were analyzed using PASW (Predictive Analytics Software) Statistics 10.0.0.

**Results:**

For all the variables analyzed, the correlation between radiographic detection of atheromatous plaque and the presence of cardiovascular disease risk factors was found to be statistically significant (RR>1.5).

**Conclusions:**

The presence of cardiovascular risk factors is related to the incidence of radiopaque lesions at the carotid artery bifurcation, indicating the presence of atheromatous plaque.

**Key words:**Orthopantomography, atheromatous plaque, cerebrovascular accident, diabetes, arterial hypertension.

## Introduction

Cerebrovascular accidents (CVA) are the third main cause of death in the industrialized world after ischemic cardiopathy and cancer. For this reason, CVA is considered one of the most important public health issues because of its high incidence and the subsequent high cost of physical and psychological rehabilitation (1).

Arterial stenosis caused by the presence of atheroma at the bifurcation of the carotid artery is the most important risk factor for CVA (2). In recent years, numerous studies have aimed to discover a low-cost, reliable and noninvasive diagnostic method for detecting these atheromas. Some researchers have proposed the use of orthopantomographs (OPG) in which the presence of calcification in the carotid artery can be detected by the appearance of a nodular and heterogeneous radiopaque mass, independent of the hyoid bone and the epiglottis, and situated above, below or within the C3-4 inter vertebral disc space or at an angle of 45o to the mandibular angle (3,4).

It is important to establish the risk factors associated with CVA, together with the prevalence of atheromatous calcifications observed in OPGs, which may act as a useful indication of each of these factors. The risk factors for CVA are the same as for cardiovascular disease in general - obesity, type II diabetes, arterial hypertension, smoking, previous antecedents of cardiovascular pathology, and a sedentary lifestyle, among others. Various published research articles have set out to establish the relation between the presence of these risk factors and the frequency of radiographic atheroma detection from OPGs, with differing results.

The aim of this study was to correlate the presence of carotid calcifications observed in orthopantomographs with the risk factors for cerebrovascular accident (previous strokes, arterial hypertension, or diabetes), recorded in patients’ medical histories.

## Patient and Method

- Study design and context:

The method used in this study followed (STROBE) recommendations (5). This observational study of cases and control subjects reviewed the database of medical histories of the Buccal Surgery and Implant Dentistry Service at the Virgin de la Paloma Hospital (Madrid, Spain). The study analyzed a total of 1,602 orthopantomographs taken between January 2010 and February 2014. The study was approved by the ethical committee of the Virgin de la Paloma Hospital (Madrid, Spain), and informed consents were signed by all participants.

- Participants.

- Inclusion criteria

All radiographs of patients younger than 18 years were excluded, as well as those considered non-evaluable, either because the orthopantomograph did not show the anatomical area of interest, or because poor technical quality prevented adequate diagnosis. Inclusion criteria were as follows: patients older than 18 years, with at least one orthopantomograph in their medical history in which the area captured included both carotid artery bifurcations.

- Diagnostic process of cases and control subjects 

To determine case and control subjects, the database of medical histories of the Buccal Surgery and Implant Dentistry Service at the Virgin de la Paloma Hospital (Madrid, Spain) was reviewed.

**Figure 1:**
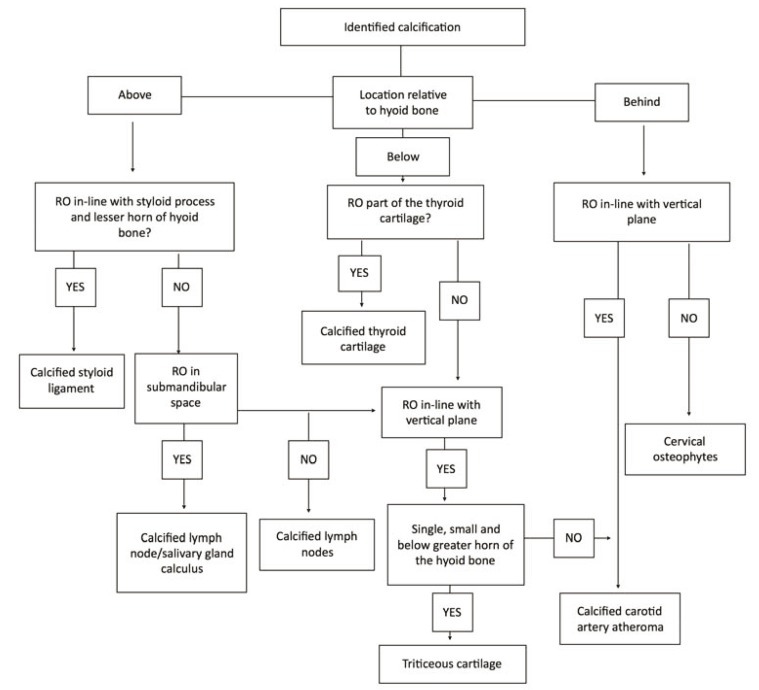
Diagram showing diagnosis of radiopaque lesions in the carotid region. Macdonald modification (6).

Cases were indicated by medical histories in which the orthopantomograph showed atheromatous plaque at the carotid artery bifurcation. All OPGs showing other calcifications in the cervical region (following the guidelines for differential diagnosis as recommended by MacDonald *et al*. (6) were excluded. Diagnosis of atheromatous plaque was defined as one or various nodular masses, situated in the soft tissues of the neck and situated 1.5 cm below and 2.5 cm posterior of the cortical edge of the midpoint of the mandibular angle, or between vertebrae C3 and C4, and at an angle of approximately 45º to the mandibular angle. (Fig. 1)

All OPGs were evaluated using the same standard negatoscope with a uniform and constant light intensity, viewed directly under the same constant ambient lighting, firstly by a recently qualified dentist, and later reviewed by a more experienced dentist who took responsibility for confirming the findings.

- Variables and data sources 

In addition to radiological examination, demographic data of each case/subject (age and sex) were registered and each medical history was reviewed to identify factors related to arteriosclerosis including: hypertension, diabetes mellitus, and antecedents of previous cerebrovascular accidents. Whether or not a patient was considered to have suffered any of these conditions was determined by diagnosis by a qualified doctor at an earlier time.

- Sample size 

The study used non-probability sampling. Epidat 3.1 statistical software established that the minimum sample size required to ensure reliability was of 135 cases and 135 control subjects.

- Statistical analysis 

The findings were entered on an Excel spread-sheet and subjected to statistical analysis using the PASW Statistics 10.0.0 program, to calculate the relative risk (RR) of the atheroma variable for each of the risk factors for cardiovascular disease registered in the subjects’ medical histories, with a 95% confidence interval. Statistical significance was set at RR>1.5.

## Results

- Participants

**Figure 2:**
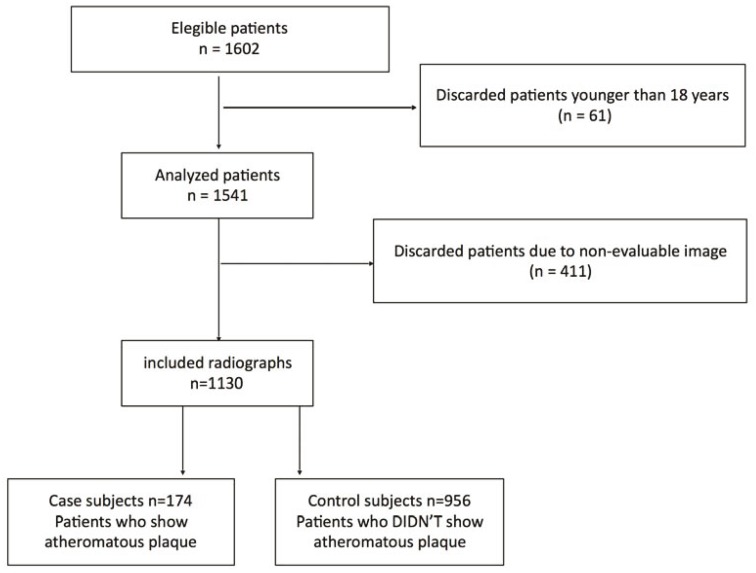
Flow diagram of patient selection process.

Figure 2 shows a flow diagram of the patient selection process.

1602 radiographs were obtained from the database of medical histories of the Buccal Surgery and Implant Dentistry Service at the Virgin de la Paloma Hospital (Madrid, Spain). 61 were discarded patients younger than 18 years, and 411 radiographs were excluded because they involved inadequate positioning or poor quality image. 1,130 medical histories were included: 174 radiographs showed the atheromatous plaque (cases) and 956 didn’t show the plaque (controls).

a) Descriptive data

The study reviewed a total of 1,130 medical histories that fulfilled the inclusion criteria. Patients were aged between 18 and 97 years, with an average age of 54.1 years and typical deviation of 21.153; 55.6% were men and 44.4% women. Specific data for each group are shown in  Table 1, Table 2.

**Table 1:**
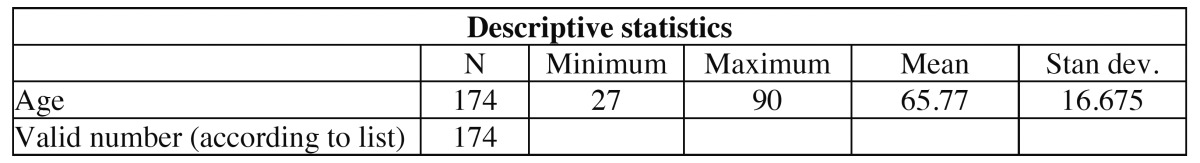
Case group, mean age (Risk factors: RF; standard deviation: Stan dev.).

**Table 2:**
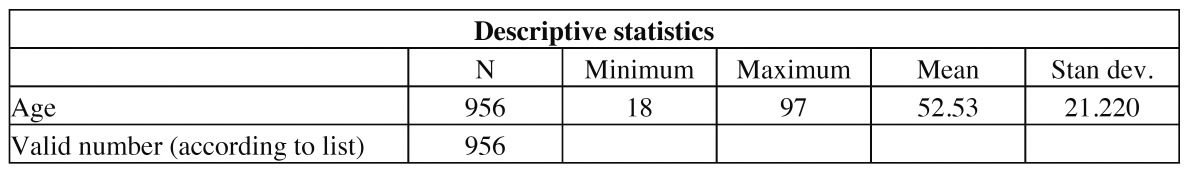
Control subjects, mean age (Risk factors: RF; standard deviation: Stan dev.).

b) Variables 

**Figure 3:**
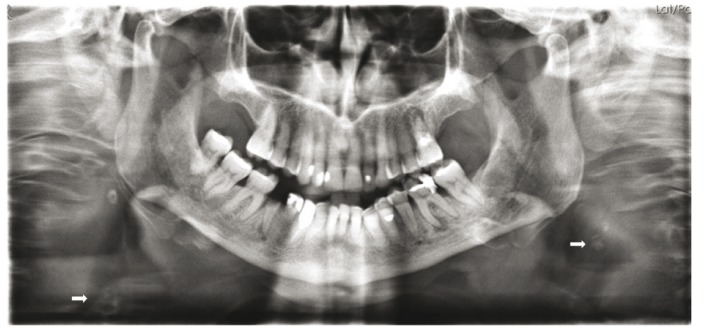
Orthopantomograph showing presence of carotid calcification on right side.

Of the 1,130 medical histories reviewed, calcifications (atheroma) were identified in 174 (15.4%), 40 in male patients and 135 in females (77.6%) (Fig. 3). The remaining 956 medical histories (84.5%) did not present calcifications (462 men and 493 women).  Table 3 shows the presence of cardiovascular risk factors in the two groups (cases and control subjects).

**Table 3:**
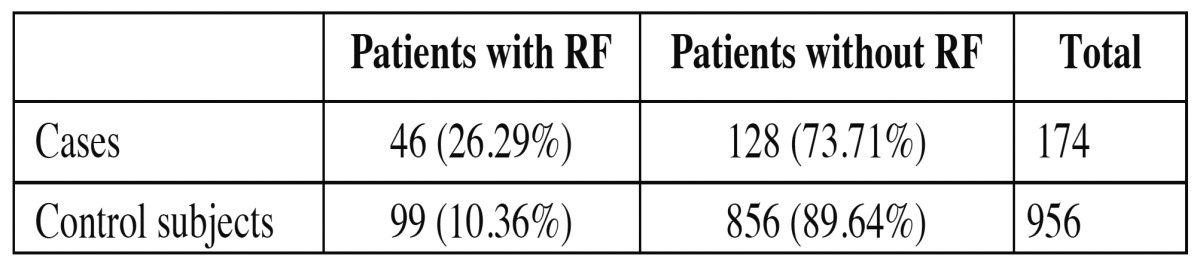
Patients presenting risk factors (RF) registered in their medical histories.

- Principal results: correlation between radiographic detection of atheromatous plaque and data obtained from medical histories 

When the correlation between the presence of atheromatous plaque in orthopantomographs and the presence of risk factors for cardiovascular disease was analyzed, all the variables showed statistically significant correlations with atheroma (RR>1.5). In this way, for patients presenting atheromatous plaque, the antecedent of having suffered a previous CVA was 9.1 (2.942-28.16) times greater than among patients who showed no radiographic atheroma. Sixteen cases of diabetes were found among the control subjects compared with nine among the cases, so the diabetes antecedent was 3.18 (1.38-7.32) times greater among patients presenting atheroma. As for hypertension, its presence was 2.35 (1.39-3.97) times greater in the atheromatous plaque group than among control subjects.

## Discussion

Although all the published research agrees that it is possible to detect atheromatous plaque from orthopantomographs, not all authors agree on the degree of its usefulness, nor on which sector or what percentage of the population might benefit from this measure. The literature review made in preparation for the present study found wide variations in the percentages of atheromatous plaque detected from OPGs. While Ertas and Sisman (7) found 66%, Bryam *et al*. (8) obtained 2.1%, while the present study found 15.5%. These differences could be due to diagnostic errors but, clearly, the chief cause is the differences between study populations (9), so that in studies with a higher number of patients presenting risk factors, the detection of calcifications is greater. This suggests that patients presenting cardiovascular risk factors will benefit from this diagnostic measure.

All authors agree on the need to perform carotid Doppler testing, not only to confirm the presence of atheromatous plaque but also to determine the degree of carotid stenosis produced in the artery, given that OPG diagnosis – although it will detect atheroma - cannot detect differences in bloodstream characteristics (9). But authors also disagree on this point. While Baumann-Bhalla *et al*. (10) have evaluated the correspondence between Doppler testing and OPG diagnosis at 81.5%, Ertas and Sisman (7) claim it is only 41%. Griniatsos *et al*. (9) approached the issue from the other direction, stating that when patients who had been subjected to carotid Doppler testing that confirmed the presence of atheroma with arterial stenosis then underwent OPG examination, calcification was detected in 70% of cases.

An important point made in a number of articles of research is the difficulty of correct diagnosis, as this depends both on the anatomical location of the carotid artery bifurcation (in other words whether or not it falls within the area evaluated) and correct differential diagnosis in relation to other anatomical structures and pathologies. Among the anatomical structures that can lead to diagnostic error, of particular note are the thyroid cartilage and, even more so, triticeous cartilage that have calcified (11,12), the latter being a source of confusion among as many as 79% of clinicians (11). The remaining anatomical structures that need to undergo differential diagnosis include: hyoid bone, calcified stylohyoid ligament, calcified stylomandibular ligament, and styloid process (13), but these structures are more easily distinguishable due to their morphological characteristics and location (14). As for pathologies, differential diagnosis must be performed to distinguish calcified lymph nodes, sialolithiasis of the submandibular glands, and free bodies (13). In this way, the main obstacles to diagnosis are incorrect identification of lesions and failure to differentiate between these and other structures or pathologies. For this reason, it is essential that dentists familiarize themselves with the necessary information and procedures given that, to date, most practitioners do not carry out examinations of this type (13).

Regarding risk factors associated with the presence of atheromatous plaque detected in OPGs, these coincide with the general risk factors for cardiovascular disease. According to the literature, the main factors are smoking, CVA antecedents, diabetes and previous cardiovascular pathology.

Both smoking in general, the number of cigarettes per day, and the years of regular smoking influence this factor significantly (7,12,15). However, the present study could not evaluate this parameter as these data were not present in all the medical histories reviewed.

As for cerebrovascular accident antecedents, Griniatsos *et al*. (9) found a higher frequency of symptomatic atheromatous plaque (with statistical significance) in patients with CVA antecedents and concluded that patients with this risk factor as well as calcifications detected in OPGs suffered an increased risk of further episodes. However, due to the study’s characteristics, the authors do not state the time passed between the cerebrovascular event and the point in time when it becomes possible to detect atheromatous plaque.

Coinciding with the present work’s results for the diabetes risk factor, Friedlander *et al*. (16) concluded that calcifications are found more frequently in OPGs of patients with type II diabetes compared to those not suffering diabetes, but the authors failed to establish a statistically significant finding in relation to the size or thickness of the calcifications. Griniatsos *et al*. (9) concluded that of all the risk factors evaluated, diabetes was the least meaningful, as it coincided with the detection of calcifications less frequently than other factors.

Regarding cardiovascular pathology antecedents, various authors state that hypertension is the most frequently occurring risk factor in patients showing atheromatous plaque in OPGs; this agrees with the present findings (4,7,10). Another study included hypertension in the group of patients with previous cardiovascular disease, along with hyperlipidemia and chronic heart diseases (17).

Other risk factors named by various authors are: the presence of renal disease (7,15), periodontal disease (18,19), neck radiation (3,15), the presence of hyperlipidemia (7,9), and cardiovascular disease (9).

Obha *et al*. (20) found statistically significant differences between men and women (the latter being more susceptible to atheromatous plaque), as in the present study in which 77.14% of patients presenting atheroma were women. Other authors (4,7-10) have not found differences in the incidence of detection between the sexes and the difference reported by Obha *et al*. (20) could be attributed to the fact that their study included 50% more female patients than male, although in the present work 55.6% of the subjects were men and 44.4% women. Obha *et al*. (20) are also the only researchers to find a higher incidence of calcification in the left artery than the right, while others agree that there are no statistically significant differences between left and right (4,7-10).

On the basis of the present findings, it can be stated that patients with calcifications in the carotid area detected from orthopantomographs suffer a greater risk of cardiovascular disease. However, as in any retrospective review of cases and control subjects, it could be that the findings suffer a degree of bias, mainly as a result of medical histories’ failure to register all the risk factors suffered by some patients. To confirm the results obtained it would be necessary to carry out a cohort study with long-term follow-up, and to support OPG findings with carotid Doppler tests.
